# Strategies to increase immunization coverage of tetanus vaccine among women in Sub Saharan Africa: a systematic review

**DOI:** 10.11604/pamj.supp.2017.27.3.11535

**Published:** 2017-06-22

**Authors:** Marius Zambou Vouking, Carine Nouboudem Tadenfok, Jean Marie Edengue Ekani

**Affiliations:** 1 Center for the Development of Best Practices in Health, Yaoundé Central Hospital, Henri-Dunant Avenue, Messa, Yaoundé, Cameroon; 2 Regional Unit of Expanded Programme on Immunization Centre Regional Delegation of Public Health, Yaoundé, Cameroon; 3 University of Buea, Department of Microbiology, Buea, Cameroon; 4 Ministry of Public Health, Yaoundé, Cameroon

**Keywords:** Strategies, immunization coverage, tetanus vaccine

## Abstract

World Health Organization (WHO) estimated in 2013 that 49,000 deaths all over the world were caused by neonatal tetanus. Only as recently as the year 2000, neonatal tetanus was a public health problem in 59 countries, but since then it has been eliminated in 36 of the countries concerned. The objective of this piece of work, therefore, was to investigate which strategies intended to increase demand for vaccination are effective in increasing anti-tetanus vaccination coverage of women in Sub Saharan Africa. We searched the following electronic databases from January 1989 to July 2016: Medline, EMBASE (Excerpta Medica Database), The Cochrane Library, Google Scholar, CINAHL (Cumulative Index to Nursing and Allied Health Literature), WHOLIS (World Health Organization Library Database), LILACS (Latin American and Caribbean Literature on Health Sciences) and contacted experts in the field. There were no restrictions to language or publication status. All study designs that could provide the information we sought were eligible, provided the studies were conducted in sub-Saharan Africa. Critical appraisal of all identified citations was done independently by two authors to establish the possible relevance of the articles for inclusion in the review. Our search strategy yielded 191 records and after assessment for eligibility, 6 papers met the criteria for inclusion. In Ivory Coast, after reorganization, health workers said they were satisfied with the work environment and the care provided in 91% and 96% of cases, respectively. In Kenya, the main factors contributing to having sufficiently immunized part of the population against tetanus are lower birth order, higher household wealth index, women's employment, making joint health-related decisions with a partner, and higher number of antenatal care visits. Particularly in Ethiopia, compared with other member countries, the size of the unimmunized population, reporting quality, fragileness of the health system, resource limitation, and others deserve further concerted attention. In Nigeria, the prevalence of missed opportunities was 66%. The factors responsible for missed opportunities were; poor history taking, lack of knowledge of the current immunization schedule, dependence on physician referral for immunization and inefficient immunization records keeping system. In Nigeria, socio-logistic variables found to be important in Expanded Programme on Immunization implementations included scheduling, health staff attitude, intersectoral collaboration, and health education. Lack of community participation was also found to be a crucial constraining factor. There are many challenges to increase immunization coverage of tetanus vaccine for women. So far very few interventions addressing these challenges have been evaluated scientifically. Community mobilization interventions to change or impact beliefs and attitudes of women are absolutely needed. Additionally, improving accessibility, affordability, availability and accommodation of vaccination service venues will make them more attractive.

## Introduction

Neonatal tetanus has been for several years a major cause of childhood mortality in developing countries [1]. In 1997 an estimated 277,376 neonatal deaths were attributed to tetanus, corresponding to a global mortality rate of 2.1 per 1000 live births [1]. More recently, as a consequence of successful vaccination programs and single-dose antenatal tetanus immunization prevention strategies, the last available worldwide World Health Organization (WHO) estimate for deaths caused by neonatal tetanus (year 2013) was 49,000 [2,3]. Although these data represent a strong reduction in disease incidence, deaths due to neonatal tetanus in 1993 represented 14% of the global causes of neonatal mortality [4] and neonatal tetanus was still responsible for about 1% of deaths that occurred among newborns worldwide in 2013 [2]. Despite impressive progress, the goal of eliminating neonatal tetanus by 2005 was later shifted to 2015 [5]. Significant progress has been made in recent years but by March 2015, neonatal tetanus remained a major public health problem (i.e. with an incidence rate of at least one neonatal tetanus case per 1000 live births at district level) in 23 countries [6]. Only as recently as 2000, neonatal tetanus was a public health problem in 59 countries, but since that time it has been eliminated from 36 countries of the concerned countries [6].

Interventions to improve vaccination outcomes are commonly grouped into those targeting health services delivery or supply (e.g. improving human resources training, logistics, cold chain maintenance and vaccine storage, effective financing, monitoring and evaluation and supportive supervision) and those that stimulate demand for vaccines. The most recent review considered shows that much remains to be done to improve immunization coverage in women [7]. We carried out a systematic review to evaluate the effectiveness of interventions that can increase immunization coverage of tetanus vaccine in women in Sub Saharan Africa. Our objectives were to assess the effect of interventions on vaccine coverage and to identify which strategies are effective. Since 1989, when the World Health Assembly called for the global elimination of neonatal tetanus, 104 developing countries out of 161 have successfully eliminated the disease [6].

## Methods

**Type of studies:** we included randomized controlled trials, controlled before and after, uncontrolled before and after, interrupted time series, cross- sectional studies, cohorts, and case control studies.

**Search strategy:** we searched the following electronic databases from January 1989 to July 2016: Medline, EMBASE (Excerpta Medica Database), The Cochrane Library, Google Scholar, CINAHL (Cumulative Index to Nursing and Allied Health Literature), WHOLIS (World Health Organization Library Database), LILACS (Latin American and Caribbean Literature on Health Sciences) and contacted four experts in the field. Search terms were combinations of “interventions”, “programs”, “approaches”, “subsidies”, “knowledge translation”, “vouchers”, “vaccination”, “immunization”, “vaccines”, “women”, “pregnant women”, and “sub Saharan Africa”. There were no restrictions to language or publication status. Our search was limited to the last seventeen years, as they match the period of enactment of the World Health Assembly’s call for the elimination of neonatal tetanus [5].

**Study design:** all study designs were eligible for inclusion provided information was on immunization coverage of tetanus vaccine in women in Sub Saharan Africa.

**Data extraction:** from each study, two authors (MZV and CNT) independently extracted data on study design, aims, location, population, intervention, follow up period and outcomes, using a pre-defined template. Disagreements were resolved by consensus or by arbitration of a third review author (JMEE). We adopted the original study definitions of comparator or control groups. We pilot tested the template on a subset of studies. In addition to vaccine outcomes, information on equity and economic outcomes were extracted. Together, three authors cross-checked and verified these data. Study authors were contacted for clarification if data were missing or unclear.

**Data extraction and management:** we retrieved full text copies of the articles identified as potentially relevant by either one or both review authors. Where appropriate, we contacted study authors for further information and clarification. The flow of study selection is described in a Preferred Reporting Items for Systematic Reviews and Meta-Analyses (PRISMA) diagram [7]. Data are reported in a narrative manner.

## Current status of knowledge

**Figure 1:**
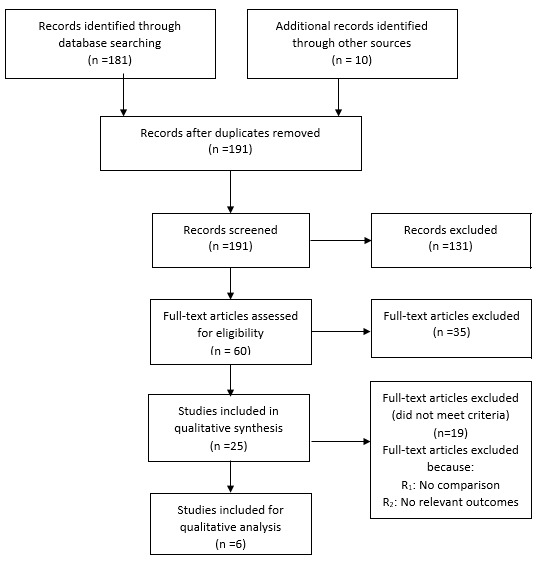
PRISMA flow diagram

**Results of the search:** searches were conducted in July 2016 and identified 191 titles (Figure 1). Studies were reviewed for relevance based on: study design, types of participants, exposures and outcomes measures. Disagreements were resolved by discussion. Six full-text articles were closely examined by authors, including a trial, identified by checking references.

**Study inclusion:** the six studies were published between 1994 and 2015; one study in Ivory Coast [8], one in Kenya [9], one in Ethiopia [10] and three in Nigeria [11-13].

**Interventions:** of the 06 studies, one study evaluated the effects of reorganization of health services on antenatal care activities [8], one study examine factors associated with sufficient tetanus toxoidimmunization among postpartum women [9], one study assessed the discourse-recourse piece of work aimed at flagging the optimization perspectives on the basis of readily available information [10], one study determine the magnitude of and the reasons for missed opportunities to immunize with tetanus toxoid at a tertiary health institution [11], one study analyze the determinants of tetanus toxoid immunization of parturient women [12], and finally, one study identified some of the factors that affected the implementation of the Expanded Program on Immunization (EPI) [13].

### The effects of reorganization of health services on antenatal care activities

In Ivory Coast, after reorganization, health workers said they were satisfied with the work environment and the care provided in 91% and 96% of cases, respectively [8]. These results were confirmed by all pregnant respondents (100%) attending the health facility (Marcory General Hospital in Abidjan), who said they were satisfied with the quality of care received [8]. This could explain the antenatal care 4 coverage rate, which increased from 39% in 2010 to 56% in 2012 and tetanus vaccination coverage which increased from 59% to 87%, although the waiting time was still too long [8].

To addressing health system challenges (inadequate infrastructure, lack of health workers), consideration should be given to individual, family and community factors that explain why vaccines are not received or administered at the appropriate time. A systematic review showed that an increase in the number of antenatal care may improve antenatal care coverage and health outcomes in low and middle income countries through reduced perinatal mortality and occurrence of low birth weight [14].

### Factors associated with sufficient tetanus toxoidimmunization among postpartum women

In Kenya, population based secondary data analysis was conducted using de-identified data from the 2008-2009 Kenyan Demographic and Health Survey for 1,370 female participants who had a live birth during or within 12 months of the cross-sectional Survey. The main factors contributing to having been sufficiently immunized against tetanus were lower birth order, higher household wealth index, women´s employment, making joint health-related decisions with a partner, and higher number of antenatal care visits [9].

Evidence show that it may be possible to successfully engage communities in different types of interventions to tackle potential weak links in the causal chain [15]. Other sectors have successfully engaged communities to design, implement and monitor development processes. Co-management, where communities are actively involved in project design, implementation, monitoring and evaluation, is integral to the success of an intervention [15].

Communication is an important component of vaccination programs, alongside service delivery, logistics, vaccine supply and surveillance and may also provide opportunities for other health promotion messages and activities [16]. The ‘Communicate to vaccinate (COMMVAC)’ project acknowledges the important role of communication in health and aims to clarify and build upon the available evidence of communication interventions to improve vaccination uptake in low and middle income countries [17].

In Ethiopia, without underestimating the progresses and successes registered so far, there are many areas that warrant further discourse and/or recourse. Compared with other member countries, the size of the unimmunized, reporting quality, fragility of systems, weak capacity, resource limitation, and others in particular with respect to Ethiopia deserve further concerted attention [10]. In Nigeria, the prevalence of missed opportunities was 66%. The factors responsible for missed opportunities were poor history taking, lack of knowledge of the current immunization schedule, dependence on physician referral for immunization and inefficient immunization record keeping system [11].

### The determinants of tetanus toxoid immunization of parturient women

A community-based study carried out on tetanus toxoid immunization status of parturient women showed a complete, partial and no coverage status of 41.2, 17.0 and 41.8 per cent respectively of women surveyed [12]. Formal education to the secondary school level was very strongly associated with complete coverage status (p < 0.001) [12]. Also of importance was the peculiar geographical terrain of the state since the place of obstacles as a negative factor was significantly more pronounced among the riverine community (p <0.001) [12]. Generally, communities in the state will require more logistics support than elsewhere in the country for any intervention measure to have an appreciable impact and on the long term the putting in place of measures aimed at raising the literacy level of the population as a whole will bring about overall improvement in the vaccine coverage in the state [12].

Researchers have observed that women´s lower social status negatively affects access to vaccines due to their weak decision-making power over resources and lack of autonomy [18]. This is why immunization coverage could be improved in some contexts by involving fathers and communities in immunization activities. Controlling the social determinants of immunization coverage is an important aspect that enables decision-makers and health program managers to improve and refine immunization program strategies [18].

As a result, the multiple roles of women (productive, maternal and social role) are often not taken into account in the design of vaccination programs. Social, time, and missed opportunity costs are borne almost exclusively by women. Women continue to be responsible for maintenance of family and household: they spend a good number of hours per day gathering water or fuel, preparing food or taking care of children and/or visiting family or helping neighbours [19].

Time constraints due to competing obligations pose significant access barriers for women and children under their care to health services. This is aggravated if a woman and her children needs are subordinated to the needs of other household members who control much of a woman’s time or restrict her ability to decide freely over how to set priorities [20, 21].

### Factors that affected the implementation of the Expanded Program on Immunization (EPI)

In Nigeria, socio-logistic variables found to be important in Expanded Programme on Immunization (EPI) implementations included scheduling, health staff attitude, intersectoral collaboration, and health education. Lack of community participation was also found to be a crucial constraining factor. As community participation/involvement is critical in sustaining health programs, social marketing techniques are suggested for future use [13].

National Vaccine Advisory Committee (NVAC) identified five major areas of opportunity to strengthen maternal immunization programs and increase uptake of recommended vaccines among pregnant women [22]. These areas for action include: 1) Enhance communication to address the safety and effectiveness of all currently recommended immunizations during pregnancy; 2) Maximize obstetrical care provider recommendation and administration of recommended maternal immunizations; 3) Focus efforts to improve financing for immunization services during pregnancy and postpartum; 4) Support efforts to increase the use of electronic health records and Immunization Information Systems among obstetrical care providers; 5) Recognize and address current vaccine liability law barriers to optimize investigations and uptake of recommended and future vaccines during pregnancy.

### Limitations

A formal meta-analysis was not feasible for this topic. Most of the studies were descriptive in nature; and none of the review studies was found to be methodologically stronger according to our quality ratings. While a meta-analysis would be meaningless in the face of such disparate studies, it is nonetheless revealing that key themes were consistent across continents, populations, and study designs, suggesting their robustness. Some of the data presented in this study were absolute numbers and in systematic reviews such vote counting is known to frequently bias the interpretation of findings, since this ignores the effect size and sample size of the studies. However, such counting is mainly used to categorize the study characteristics not to report any study outcomes as such.

## Conclusion

Addressing these challenges have been evaluated scientifically. The review has shown that there are many challenges to increasing immunization coverage of tetanus vaccine in women and central to these challenges is the level of Community Mobilization Interventions and the attitude of women. So far very few interventions are made and women’s attitude hinder progress. In this regard, community mobilization interventions to impact change in the beliefs and attitude of women are absolutely needed. In addition, improving accessibility, affordability, availability and accommodation of vaccination service venues will make them more attractive to the targeted population and hence impact coverage.

### What is known about this topic

- Interventions to improve vaccination outcomes are commonly grouped into those targeting health service delivery or supply;
- The most recent review considered shows that much remains to be done to improve immunization coverage in women.

### What this study adds

- Community mobilization interventions to improve beliefs and attitudes of women are absolutely needed to improve coverage of tetanus vaccine in women in Sub Saharan Africa;
- Controlling the social determinants of immunization coverage is an important aspect that enables decision-makers and health program managers to improve and refine immunization program strategies.

## Competing interests

The authors declare no competing interests.
